# 以反复气胸合并胸腔积液为表现的胸膜高分化乳头状间皮肿瘤1例

**DOI:** 10.3779/j.issn.1009-3419.2026.101.11

**Published:** 2026-05-20

**Authors:** Zhe LI, Fanyi KONG, Bo YANG, Tianrun SONG, Shuai ZHAO, Xiang SONG

**Affiliations:** 061000 沧州，沧州市中心医院胸部肿瘤中心; Department of Thoracic Tumor, Cangzhou Central Hospital, Cangzhou 061000, China

**Keywords:** 高分化乳头状间皮肿瘤, 气胸, 病例报道, Well-differentiated papillary mesothelioma, Pneumothorax, Case report

## Abstract

高分化乳头状间皮肿瘤（well-differentiated papillary mesothelial tumor, WDPMT）是一种罕见肿瘤，具有典型的乳头状结构、温和的细胞学特征、倾向于表面扩散而无间质浸润等特点。现报告1例以反复自发性气胸伴胸腔积液为突出表现的胸膜WDPMT患者的诊疗经过，并对WDPMT的研究现状进行文献回顾。患者女，58岁，因反复性自发性气胸伴胸腔积液7年，胸闷、气短2周就诊。既往7年自发性气胸伴胸腔积液复发4次，患者自述无石棉暴露史。胸腔镜探查见肺大疱形成，合并胸膜增厚、胸腔积液与积气，脏层及壁层胸膜表面散在灰白色结节。胸膜结节组织活检病理诊断为WDPMT。综合该病例特征并复习相关文献，对于反复发生自发性气胸且伴有胸腔积液的患者，临床医师需警惕WDPMT可表现为此类非典型临床过程。

## Abstract (en)

Well-differentiated papillary mesothelial tumor (WDPMT) is a rare neoplasm characterized by a distinct papillary architecture, bland cytological features, and a propensity for superficial spread without stromal invasion. We herein report the diagnosis and management of a patient with pleural WDPMT presenting predominantly with recurrent spontaneous pneumothorax and pleural effusion, along with a review of the current literature on this entity. The patient was a 58-year-old female who presented with a two-week history of chest tightness and dyspnea on a background of recurrent spontaneous pneumothorax and pleural effusion over the preceding seven years. She had experienced four episodes of recurrence during that period and denied any history of asbestos exposure. Thoracoscopic exploration revealed pulmonary bullae, pleural effusion and pneumothorax, pleural thickening, and scattered grayish-white nodules on both the visceral and parietal pleura. Histopathological examination of the pleural nodule biopsy confirmed the diagnosis of WDPMT. Based on the features of this case and a review of the literature, clinicians should be aware that WDPMT may present with the atypical manifestation of recurrent spontaneous pneumothorax accompanied by pleural effusion.

高分化乳头状间皮瘤（well-differentiated papillary mesothelioma, WDPM）是一种罕见的上皮样间皮瘤亚型，好发于育龄期女性的腹膜，其次可见于胸膜、心包膜及睾丸鞘膜等部位^[1]^。2021年世界卫生组织（World Health Organization, WHO）胸部肿瘤分类将“WDPM”正式更名为“高分化乳头状间皮肿瘤”（well-differentiated papillary mesothelial tumor, WDPMT），并被归为良性或浸润前病变组，与腺瘤样肿瘤、原位间皮瘤并列^[2]^。胸膜型WDPMT的临床表现缺乏特异性，患者多以胸腔积液为主要表现，可伴有咳嗽、胸闷、呼吸困难等症状，本文报道1例以反复自发性气胸伴胸腔积液为主要表现的胸膜型WDPMT病例，据文献检索，既往以自发性气胸伴胸腔积液为主要表现的WDPMT报道较少，为加深对该疾病及其不典型临床表现的认识，现对本例患者的临床特征、诊断过程进行总结，报道如下。

## 1 病例资料

患者女，58岁，2025年12月25日因反复性自发性气胸7年，胸闷、气短2周就诊于沧州市中心医院，无咳嗽、咳痰，无痰中带血，无胸背部疼痛，无发热、盗汗等症状。患者7年前因右侧胸腔积液伴积气在外院行胸腔闭式引流治疗后未见明显好转就诊于我院，行胸腔镜手术，术中见胸膜结节及肺大疱，术后病理：（右肺大疱）肺大疱，并见乳头状间皮细胞增生结节，倾向WDPMT。随诊复查。2年前因双侧自发性气胸于我院行胸腔镜下左肺大疱切除术，8个月前患者再次因反复胸腔积液伴积气行“右肺大疱切除+胸膜剥脱术”，病理再次证实为WDPMT。6个月前发现盆腔团块，行开腹全子宫+双侧附件切除术+大网膜切除术+阑尾切除术+盆腔淋巴结清扫术+双侧骶韧带肿物切除+直肠前壁肿物切除术。术后病理提示WDPMT，与原发胸膜病变一致。半个月前再次出现右侧胸腔积液伴积气行胸腔闭式引流治疗，引流效果差，为求进一步治疗就诊于我院。既往体健，无吸烟、饮酒史，无肿瘤家族史，否认有石棉接触史。查体：双锁骨上区未触及肿大淋巴结。右侧胸部叩诊呈鼓音，左侧叩诊呈清音；右侧呼吸音减弱，左侧呼吸音清晰；右侧呼吸动度减弱，左侧呼吸动度正常。心脏及腹部查体未见明显异常。胸部计算机断层扫描（computed tomography, CT）显示右侧胸膜增厚，右侧胸腔积液减少，积气增多，右侧胸腔纤维条索影，见肺气肿及肺大疱（图1）。充分向患者家属交待病情，患者反复性气胸发作，于2025年12月29日在全麻下行右肺大疱切除+胸膜剥脱术，术后病理：（右侧胸膜）检材为纤维脂肪及横纹肌组织，表面衬覆间皮细胞增生，局部乳头样增生，既往WDPMT病史，考虑具有肿瘤发展趋势。（右肺大疱）肺被膜表面局灶间皮细胞乳头样增生。（右肺上叶组织）肺被膜表面小灶间皮细胞乳头样增生（图2A）。免疫组化：Calretinin（+），CK7（+），CK5/6（局灶+），MC（+），Ki-67（低表达，1%-2%），PAX8（部分+），P53（散在+），TTF-1（-），Desmin（-）（图2B-2D）。术后3个月随诊复查患者胸腔未见明显积气积液，未诉明显不适。

**图1:**
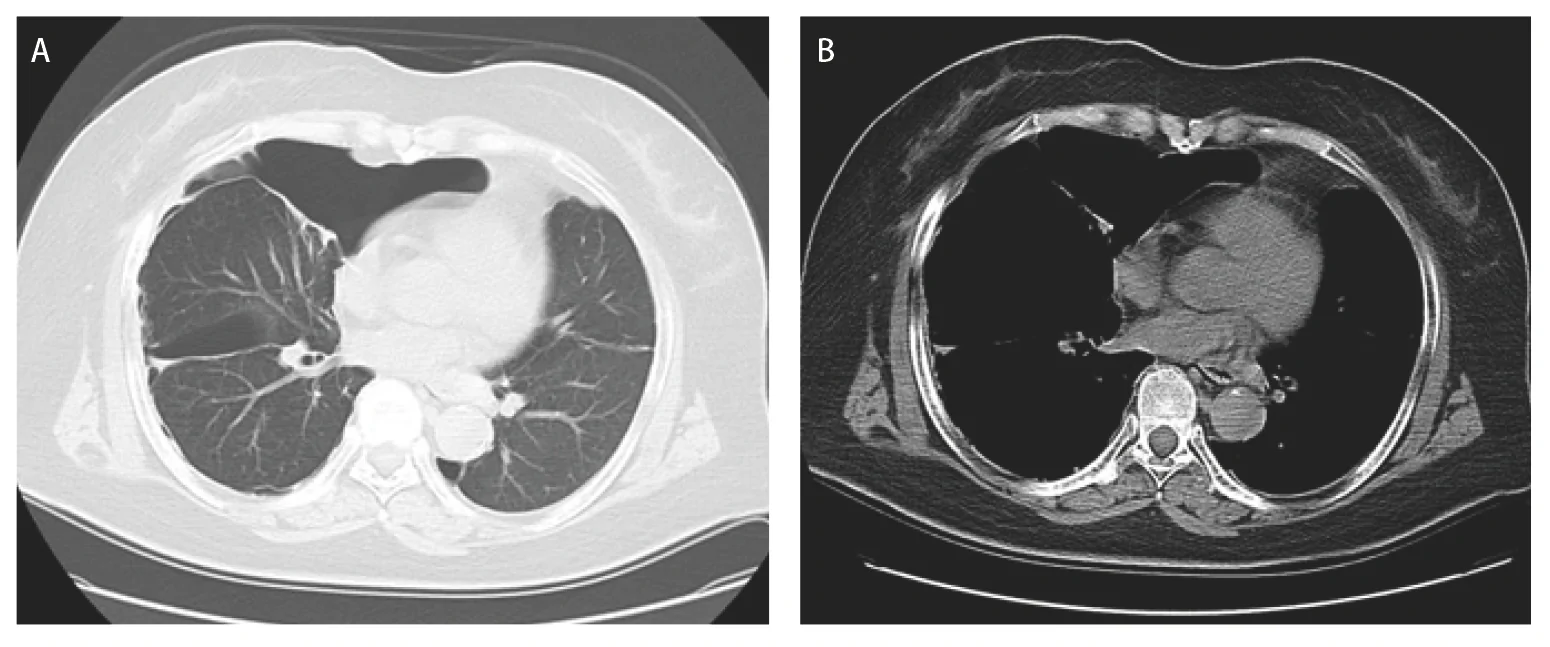
胸部CT平扫提示右侧胸膜增厚，右侧胸腔积液减少、积气增多，右侧胸腔纤维条索影。可见肺气肿及肺大疱。A：手术前胸部CT检查（肺窗）；B：手术前胸部CT检查（纵隔窗）。

**图2:**
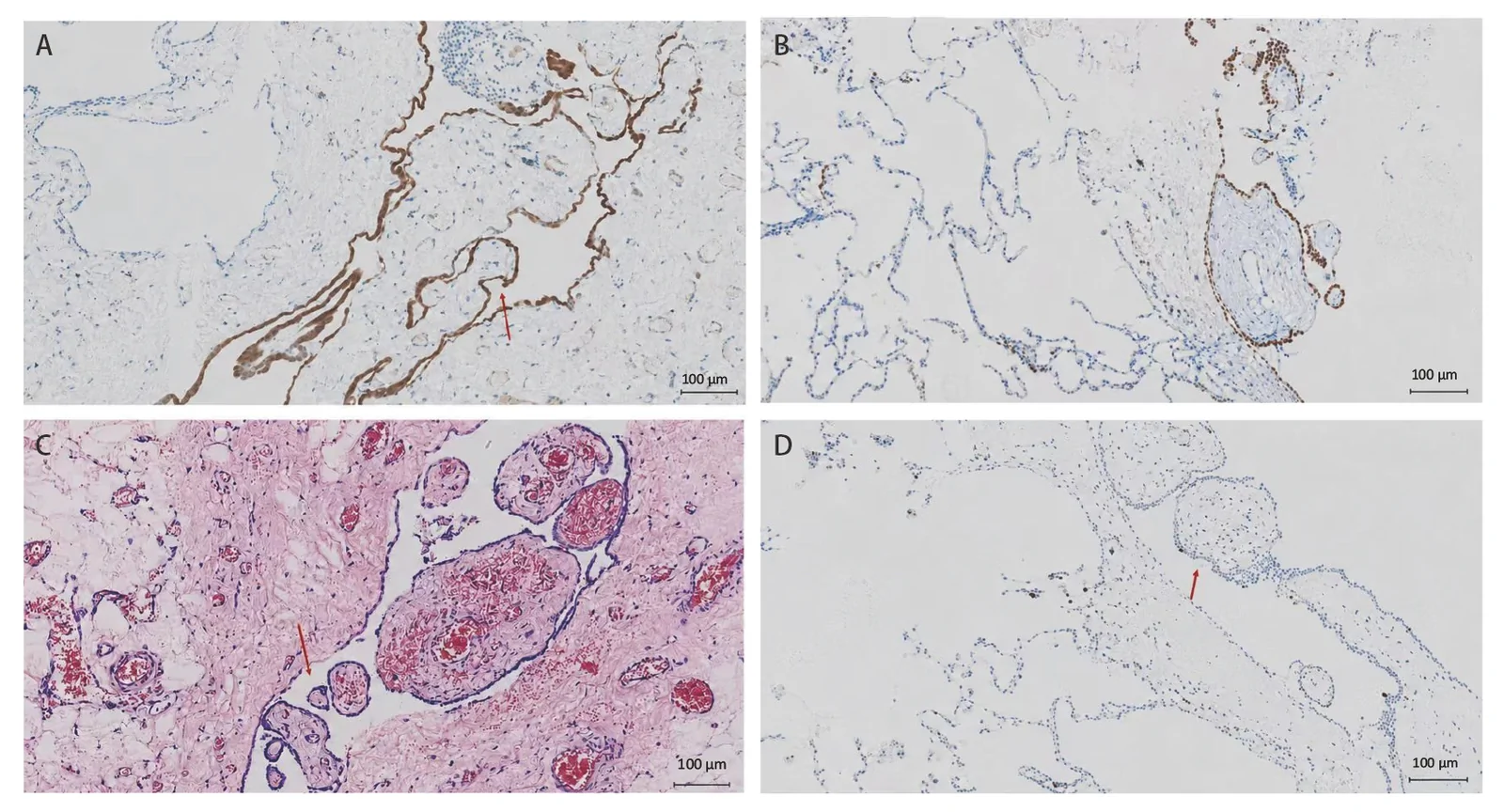
病理HE染色。A：（右侧胸膜）检材为纤维脂肪及横纹肌组织，表面衬覆间皮细胞增生，箭头指示局部乳头样增生；B：CK5/6免疫组化结果；C：Ki-67免疫组化结果（箭头指示低表达，1%-2%）；D：PAX8免疫组化结果（箭头指示部分+）。

该病例报道已取得患者知情同意。本病例报道获得沧州市中心医院伦理委员会批准（伦理审批项目号：2025-032-01）。

## 2 讨论

WDPMT是一种罕见的间皮肿瘤，由具有乳头状结构的间皮细胞组成。由于其生物学行为惰性，患者生存期较长，该肿瘤预后通常较良好^[3]^，但该肿瘤常伴其他肿瘤或反应性增生，极易与一些形态相似的肿瘤混淆，造成诊断上的困难。

WDPMT发病率占所有间皮肿瘤的0.3%-1.5%^[4]^，其好发于腹膜，且发病年龄广泛，多见于育龄期妇女^[3]^，女性与男性的比例约为5:1。腹膜WDPMT多为手术时偶然发现，患者可能无症状或仅有轻微的腹部不适，病变腹膜或浆膜表面见单个小结节或多个粟粒样结节^[5]^。相比之下，胸膜WDPMT更为少见，文献^[6]^报道的病例不足100例，与腹膜WDPMT不同的是，其在性别分布上无明显差异，且发病年龄较腹膜WDPMT更广，从儿童到老人均有报道。在临床表现上胸膜WDPMT缺乏特异性，主要以胸腔积液、胸痛、呼吸困难或体检偶然发现的胸膜结节为特征^[7]^。影像学检查方面，CT扫描可显示胸膜增厚、胸膜结节或胸腔积液，但早期病变尤其是微小病灶常难以通过常规影像学方法检出，正电子发射型计算机断层显像（positron emission computed tomography, PET）/CT在评估病变范围和排除远处转移方面具有一定价值，但对于胸膜WDPMT的诊断特异性和敏感性仍有待进一步研究。对于复发性胸腔积液而常规影像学检查未能明确病因的患者，外科胸腔镜检查在获取胸膜组织标本方面具有重要价值，尤其适用于CT难以发现的微小病变患者^[8]^。本例患者以反复性自发性气胸为突出临床特征，CT检查提示胸膜增厚并胸腔积气。尽管间皮瘤可致胸膜增厚及脏层胸膜结构异常，但以反复性自发性气胸伴胸腔积液为主要表现者，既往文献报道尚不多见。该非典型临床表现提示临床医师在处理反复发作性气胸患者时，尤其对于合并胸腔积液或胸膜增厚者，应高度警惕胸膜WDPMT的可能。

胸膜WDPMT的生物学行为总体呈惰性，但并非完全良性。文献^[9]^报道显示，胸膜WDPMT的复发率为30%-50%，多发生于术后2-10年，部分病例可多次复发，虽然复发率较高，但WDPMT患者的总生存率良好，5年生存率可达90%-100%，10年生存率为85%-90%，其中少数病例（2%-5%）可发生恶性转化为弥漫性恶性间皮瘤，这类患者预后显著变差，中位生存期仅12-18个月^[2]^。影响预后的因素包括：肿瘤侵袭深度、细胞异型性、核分裂象数量、Ki-67增殖指数和是否完全切除^[10]^。本例患者因WDPMT相关并发症共经历4次手术治疗，反映了该肿瘤不容忽视的局部复发倾向。更需警惕的是，患者于初次术后6年出现盆腔转移，免疫组化证实为间皮源性，这一罕见事件提示该例WDPMT可能已偏离惰性生物学行为，呈现侵袭性演进趋势。因此，对于此类患者，即便初诊为低度恶性潜能肿瘤，亦应建立长期密切随访机制，以早期识别疾病进展并适时调整治疗策略。

WDPMT的临床特征尚无显著特异性，影像学检查仅提供参考，而该病诊断的金标准为病理诊断。文献^[11]^报道该病具有如下病理学诊断：（1）肿瘤呈乳头状或管状乳头状结构；（2）间质内未见浸润性生长；（3）瘤细胞呈立方状，排列整齐；（4）核分裂象少见，增殖活性低；（5）细胞具单一胞核，间质内未见浸润性生长，本例患者均有上述镜下表现。免疫组化检测中，CK5/6与Calretinin是确认间皮分化的可靠标志物^[12]^，研究^[5]^显示，PAX-8在WDPMT中或具有较高的灵敏度和特异度，另有学者^[4]^表示，此类患者中Ki-67增殖指数为<1%-5%，此例患者免疫组化表现与上述观点一致。

WDPMT的治疗策略尚未标准化，目前主要基于个案报道和小样本回顾性研究。完整的肿瘤切除是首选治疗方法，对于局限性病变，单纯肿瘤切除或伴部分胸膜/腹膜切除可获得良好的局部控制，而对于病变面积较大、累及范围较广的患者，治疗策略目前尚不统一，文献^[13]^指出，WDPMT手术切除后预后良好，多数患者无需额外辅助治疗，亦有学者^[14]^认为，对于病灶呈多发性或难以根治性切除的患者，应考虑采用联合化疗方案，胸膜WDPMT的治疗策略可参考腹膜WDPMT的经验，但需注意二者在病因学和生物学行为上的差异，近年来，免疫检查点抑制剂如程序性死亡受体1/程序性死亡配体1（programmed death receptor 1/programmed death-ligand 1, PD-1/PD-L1）抑制剂在恶性间皮瘤治疗中显示出一定疗效，但在WDPMT中的应用仍处于探索阶段^[15]^。考虑到WDPMT的分子特征与恶性间皮瘤存在显著差异，未来针对肿瘤坏死因子相关受体因子7（tumor necrosis factor receptor associated factor 7, TRAF7）突变或其他特异性分子靶点的靶向治疗可能为WDPMT患者提供新的治疗选择。鉴于WDPMT潜在的惰性生物学行为，临床实践中应审慎评估手术干预的指征与获益。对于本例因肿瘤所致反复发作性气胸而多次接受手术治疗的患者，其临床经过提示，单纯依赖手术处理局部并发症可能未能充分遏制疾病的长期病程演进。尽管完整切除局限性病灶被视为此类肿瘤的理想治疗手段，然而，对于表现为复发性气胸等非特异性症状且病灶弥散或难以彻底清除的患者，反复的胸膜固定术或局限性胸膜剥脱术在实现症状控制的同时，亦可能面临较高的复发风险。因此，本文观点认为，针对胸膜WDPMT的外科决策需综合考量病灶范围、患者耐受性及远期预后。手术方案的选择应趋于保守与个体化，以解除危急症状为主要目标，避免过度追求病灶的根治性切除。在手术对症处理后，确立长期、规范的影像学随访体系至关重要，以早期识别疾病进展或恶性转化的潜在征象。对于术后症状反复或存在高危病理特征的患者，未来有必要进一步探索辅助治疗手段（如靶向治疗或免疫治疗）在延缓疾病进程、降低复发率方面的临床价值。

综上所述，WDPMT是一种罕见的间皮源性肿瘤，具有独特的临床病理学特征及相对惰性的生物学行为。本文报道了1例以反复性自发性气胸伴胸腔积液为主要临床表现的胸膜WDPMT，其非典型的起病方式丰富了临床医师对该疾病表现谱的认识。尽管WDPMT总体预后良好，但其潜在的复发倾向及向浸润性恶性间皮瘤转化的风险仍不容忽视。本病例提示，对于表现为反复发作性气胸，尤其合并胸腔积液或影像学提示胸膜异常的患者，应将WDPMT纳入鉴别诊断范畴，避免漏诊或误诊，力求实现早期识别与及时干预。
